# Intrinsic epigenetic control of angiogenesis in induced pluripotent stem cell-derived endothelium regulates vascular regeneration

**DOI:** 10.1038/s41536-022-00223-w

**Published:** 2022-05-12

**Authors:** Bria L. Macklin, Ying-Yu Lin, Kevin Emmerich, Emily Wisniewski, Brian M. Polster, Konstantinos Konstantopoulos, Jeff S. Mumm, Sharon Gerecht

**Affiliations:** 1 Department of Chemical and Biomolecular Engineering, The Institute for NanoBioTechnology, Physical Sciences-Oncology Center, Johns Hopkins University, Baltimore, MD 21218 USA; 2 Department of Ophthalmology, Wilmer Eye Institute and McKusick–Nathans Institute of Genetic Medicine, Johns Hopkins University School of Medicine, Baltimore, MD 21205 USA; 3 Department of Anesthesiology and Center for Shock, Trauma, and Anesthesiology Research, University of Maryland School of Medicine, Baltimore, MD 21201 USA; 4 Department of Biomedical Engineering, Duke University, Durham, NC 27708 USA

**Keywords:** Induced pluripotent stem cells, Regeneration

## Abstract

Human-induced pluripotent stem cell-derived endothelial cells (iECs) provide opportunities to study vascular development and regeneration, develop cardiovascular therapeutics, and engineer model systems for drug screening. The differentiation and characterization of iECs are well established; however, the mechanisms governing their angiogenic phenotype remain unknown. Here, we aimed to determine the angiogenic phenotype of iECs and the regulatory mechanism controlling their regenerative capacity. In a comparative study with HUVECs, we show that iECs increased expression of vascular endothelial growth factor receptor 2 (VEGFR2) mediates their highly angiogenic phenotype via regulation of glycolysis enzymes, filopodia formation, VEGF mediated migration, and robust sprouting. We find that the elevated expression of VEGFR2 is epigenetically regulated via intrinsic acetylation of histone 3 at lysine 27 by histone acetyltransferase P300. Utilizing a zebrafish xenograft model, we demonstrate that the ability of iECs to promote the regeneration of the amputated fin can be modulated by P300 activity. These findings demonstrate how the innate epigenetic status of iECs regulates their phenotype with implications for their therapeutic potential.

## Introduction

The advent of human induced pluripotent stem cells (hiPSCs) marked a critical moment in the field of personalized, regenerative medicine^1^. Reduction in regenerative capacity, a hallmark of diseased endothelium, highlights the dire need for efficient and robust hiPSC-derived endothelial cell (iEC) based therapies. Numerous studies have explored iEC functionality both in vitro and in vivo; however, as we continue to develop further insight into the functional capabilities of these cells, research regarding the molecular mechanisms of regeneration lags behind.

Vascular regeneration occurs via sprouting angiogenesis, which activates a competitive tip cell by exogenous cues and drives new blood vessels^2^. These activated tip cells are highly migratory, with long extended filopodia that allows them to probe and respond to their environment. They also possess high energetic needs, favoring glycolysis over oxidative phosphorylation^3^. Neighboring cells, termed stalk cells, are highly proliferative and form lumenized vessels guided by tip cells^2^. The VEGF/VEGF Receptor 2 (VEGFR2) signaling pathway is a crucial upstream mediator in these two distinct cell fates. Phosphorylation of VEGFR2 at the surface of the tip cell activates downstream Notch-mediated lateral inhibition, allowing the tip cells to dominate the angiogenesis process by competitively inhibiting tip cell specification in neighboring stalk cells^4^. In addition to regulating cell fate through Notch signaling, VEGF/VEGFR2 signaling has been shown to mediate tip cell migration by stimulating microtubule organization^5^ and increasing glycolysis within the cell by upregulating glycolytic enzymes^3,6^.

Once the new vessel is formed, the ECs become quiescent, losing their angiogenic phenotype upon upregulation of signaling pathways that maintain homeostasis, including proper barrier function, nutrient transport, and shear responsiveness in an organ-specific manner^7^. In healthy patients, quiescent ECs can switch back to an angiogenic state in response to injury or vessel damage; however, patients with impaired angiogenic capacities display poor new vessel formation. Insufficient vessel growth is a common symptom of diabetes, Alzheimer’s disease, and stroke^8^. Studies have linked cellular loss of function and lack of regenerative capacity to epigenetic changes in DNA and chromatin structure. Specifically, methylation and acetylation, resulting in condensed or loosened chromatin, respectively, have been indicated as key effectors in the aging process, with DNA methylation levels directly correlated to age and age-related outcomes^9^. Additionally, recent work demonstrated the ability to revert aged retinal ganglion cells to an embryonic state by regulating an epigenetic signature that restores axonal regenerative capacity by increasing methylation inhibitors, TET1 and TET2^10^. In the cardiovascular system, in vitro studies have revealed a unique role of histone acetyltransferases (HATs) and histone deacetylases (HDACs) in controlling essential EC functions such as differentiation sprouting angiogenesis, migration, and response to shear stress^11–14^. However, whether and how such epigenetic changes modulate the regenerative capacity of iECs has not been studied.

Here, we define a critical mechanism that regulates the angiogenic capacity of iECs. We show that iECs have a high angiogenic ability in vitro modulated by increased expression of VEGFR2. We find that this process is regulated by acetylation of histone H3 at lysine 27 (H3K27ac) by the HATs P300, resulting in increased expression of VEGFR2. Utilizing a zebrafish xenograft model, we demonstrate that iECs promote the regeneration of damaged tissues and that this capacity to enhance regeneration can be diminished by inhibiting P300 activity.

## Results

### iECs display tip cell-like sprouting and glycolytic activity

**Fig. 1:**
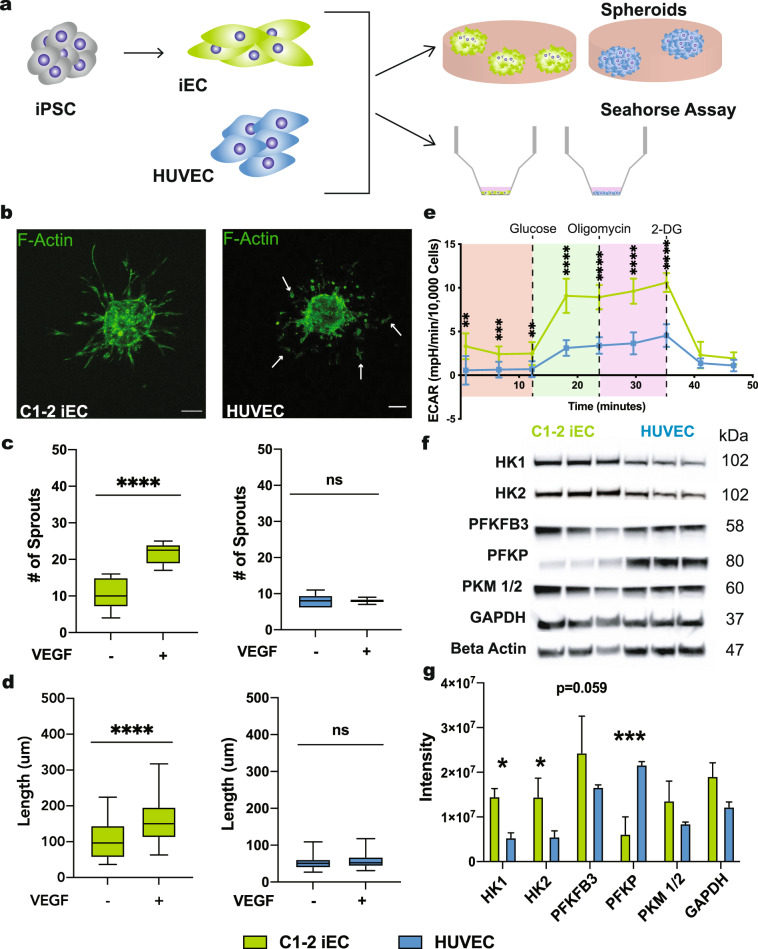
iECs display extensive sprouting and a high glycolytic rate. iECs derived from the C1-2 hiPSC line were analyzed for sprouting abilities. **a** Schematic is outlining the experimental workflow. **b** Representative confocal images of HUVEC (left) and iEC (right) sprouting spheroids after 24 h in collagen gel in media supplemented with or without VEGF. Arrows indicate disconnected cells. **c** Quantification of the average number (#) of sprouts per spheroid and **d** average sprout length (*N* = 3, *n* = 25–30). **e** Extracellular Acidification Rate measured via Seahorse Assay in C1-2 iECs and HUVECs (red, non-glycolytic acidification; green, glycolysis; purple, glycolytic max; *N* = 3, *n* = 9). **f** Western blot analysis of glycolysis enzymes and **g** quantification, *N* = 3. Statistical significance levels are set at *p ≤ 0.05, **p ≤ 0.01, ***p ≤ 0.001, and ****p ≤ 0.0001 by two-tailed Student’s t-test and Sidak’s multiple comparison test. For bar graphs: data are presented as mean ± SD. For box and wisker plots: centerline, median; box limits, upper and lower quartiles; wiskers, minimum to maximum value. Scale bar: 100 μm.

To examine iEC angiogenic capacity, we compared them to human umbilical vein ECs (HUVECs). HUVECs were chosen as a control to align with seminal studies delineating angiogenesis mechanisms using HUVECs in vitro^3,15,16^. We used both spheroids and seahorse assays to determine angiogenic capacity (Fig. 1a). To assess angiogenic sprouting in each cell type, 3D spheroids were formed over 24 h using the hanging droplet method and then embedded in collagen gels in Endothelial Cell Growth Medium (ECGM) with and without VEGF. After 24 h, we observed a substantial increase in the number and length of sprouts originating from the iEC spheroids compared with HUVEC spheroids in gels supplemented with VEGF (Fig. 1b). Under the same condition, HUVEC spheroids displayed more detached cells, suggesting these cells were migrating away from the spheroid, not sprouting (note white arrows in Fig. 1a). Quantification of only the sprouts connected to the spheroids confirmed that HUVECs failed to respond to VEGF supplementation, with no significant difference in the number of sprouts or length between conditions with and without VEGF (Fig. 1c, d). Conversely, iEC spheroids from both C1-2^17^ and 6.2^18^ hiPSC lines exhibited an increased number of connected sprouts and increased length when supplemented with VEGF (Supplementary Fig. 1a, b).

The role of glycolysis in endothelial tip cells is crucial in allowing migrating cells to produce the ATP necessary to meet energetic demands^3,19,20^. Results of the seahorse assay revealed a higher glycolytic rate in iECs as compared to HUVECs, measured as the 2-deoxyglucose (2-DG)-suppressible increase in extracellular acidification rate (ECAR) upon glucose addition that reflects glycolytic lactic acid production (Fig. 1e). To investigate further, we examined enzymes that regulate glycolysis, including HK1, HK2, PFKFB3, PFKP, PKM1/2, and GAPDH, using Western blotting. We found that iECs had higher HK1, HK2, and PFKFB3 expression than HUVECs (Fig. 1f, g). These enzymes represent key modulators of EC metabolism and function, with HK1/2 phosphorylation of glucose to glucose-6-phosphate serving as the first and rate-limiting step in glycolysis^21^ and PFKFB3 being vital in vessel sprouting^3^.

### iECs display tip cell phenotype with filopodia and migration in response to VEGF gradients

**Fig. 2:**
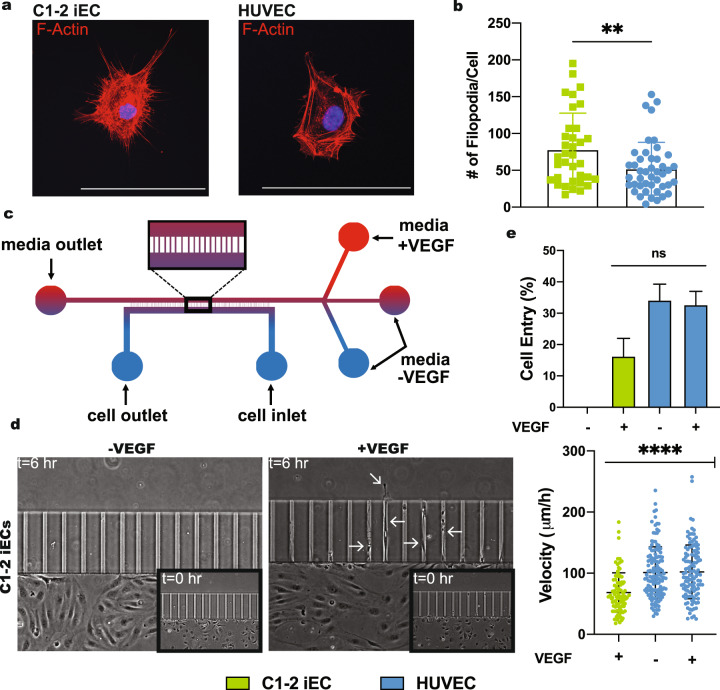
Filopodia formation and VEGF-dependent migration of iECs. **a** Filopodia formation in C1–2 iECs and HUVECs was assessed using F-actin and Myosin X stain followed by confocal microscopy of dispersed cells. **b** Quantification of filopodia was analyzed using an F-actin stain with the Image J plug-in FiloQuant. (*N* = 3, *n* = 45). Graph plotted without outliers. **c** Schematic of the microfluidic device with amplification of the 2D cell culture and migration channels region and **d** image of C1-2 iEC migration in the channels with and without a VEGF gradient at *t* = 6 h. Arrows indicate iECs migrating through the channels. The boxed images show cells at *t* = 0. **e** Quantification of cell entry and velocity in the channel of individual cells (*N* = 3, *n* = 105–120). Statistical significance levels are set at *p ≤ 0.05, **p ≤ 0.01, ***p ≤ 0.001, and ****p ≤ 0.0001 by two-tailed Student’s t-test and Tukey’s multiple comparison test. Data are presented as mean ± SD. Scale bar: 100 μm.

Endothelial tip cell migration is mediated by filopodia formation, bundled actin filaments that allow cells to migrate through and sense their environment. These filopodia guide sprouting angiogenesis via their response to VEGF gradients^5^. To assess filopodia formation and actin organization in iECs and HUVECs, cells were seeded sparsely as a single-cell suspension on coverslips and analyzed using the ImageJ plug-in FiloQuant. We found that iECs from C1-2^17^, 6.2^18^, and 1159^22^ displayed more filopodia per cell than HUVECs (Fig. 2a, b and Supplementary Fig 2a). Although HUVECs formed fewer filopodia than iECs, actin filaments in the HUVEC cytoskeleton organized into lamellipodia-like structures. Unlike filopodia-mediated migration, migration mediated via lamellipodia is random, fast, and unresponsive to chemo-attractants^23^.

To assess this differential actin organization’s role in EC migration, we utilized a microfluidic-based platform^24^ to determine the migratory phenotype and chemotactic response to a VEGF gradient. iECs or HUVECs were seeded into a 2D multichannel microfluidic device (Fig. 2c) in ECGM without VEGF. Media with or without VEGF was added to the top chamber to establish baseline and gradient conditions, respectively, and cells were allowed to migrate over 12 h. iECs in devices without a VEGF gradient did not fully migrate through the channels. In contrast, HUVECs in devices with and without a VEGF gradient were highly migratory in both conditions. A similar number of cells entered the chamber with a higher velocity than iECs (Fig. 2d, e and Supplementary Fig 2b). Time-lapse videos show that iECs extend cellular processes into the channel at early time points to probe the environment. In the presence of a VEGF gradient, iECs developed elongated cellular processes into the channels, followed by the entire cell body migrating into the top chamber (Supplementary Videos 1, 2). These results indicate that iEC migration occurs in a controlled, organized manner mediated by the cytoskeletal organization and is selectively responsive to VEGF, while HUVECs displayed no preferential migration towards VEGF.

### iECs have increased the expression of VEGFR2

**Fig. 3:**
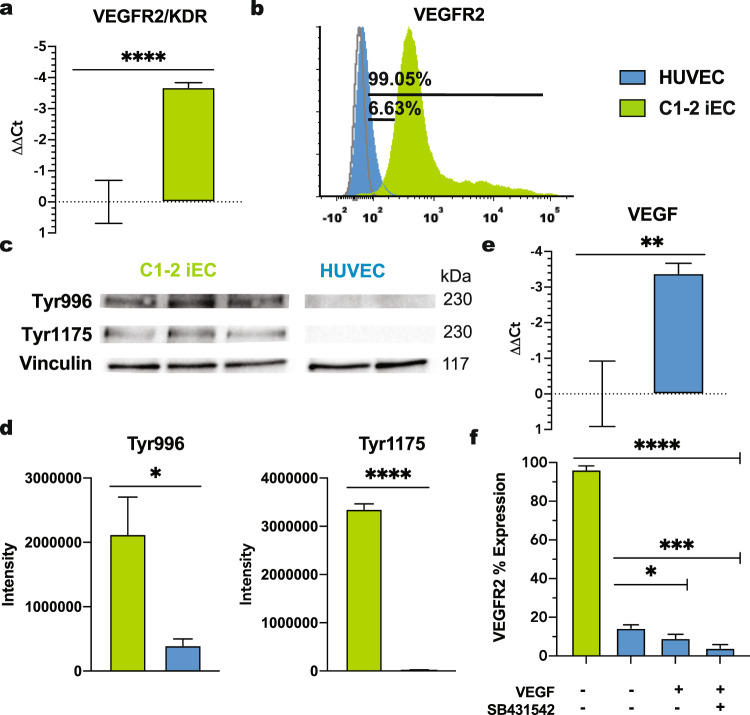
High VEGFR2 expression in iECs. **a** RT-qPCR data for VEGFR2 normalized to HUVECs. (*N* = 3; *n* = 9). **b** Representative flow cytometry data for VEGFR2 in HUVECs (blue, 6.63%) and C1-2 hiPSC-ECs (green, 99.05%) (*N* = 3). **c** Western blot analysis of VEGFR2 phosphorylation at tyrosine 996 and 1175 and **d** quantification. (*N* = 3). **e** RT-qPCR data for VEGF normalized to C1-2. (*N* = 3, *n* = 9). **f** VEGFR2 expression in HUVECs cultured with and without VEGF (50 ng/ml) and the TGFβ inhibitor, SB431542. (*N* = 3) Statistical significance levels are set at *p ≤ 0.05, **p ≤ 0.01, ***p ≤ 0.001, and ****p ≤ 0.0001 by two-tailed Student’s t-test and Tukey’s multiple comparison test. Data are presented as mean ± SD. Scale bar: 100 μm.

VEGFR2 activation has been shown to modulate angiogenesis and tip cell formation in ECs^5,25^. Therefore, we assessed VEGFR2 expression in iECs and HUVECs and found higher VEGFR2 mRNA and membrane-bound protein levels in iECs than HUVECs (Fig. 3a, b). This elevated expression of VEGFR2 was seen across hiPSC lines at both the mRNA and protein levels (Supplementary Fig 3a, b). In addition to higher expression of VEGFR2 in iECs, we also measured increased phosphorylation of Y996 and Y1175 VEGFR2 sites in iECs indictive of the activated status of the VEGFR2^26,27^ (Fig. 3c, d). To assess relative VEGF production in iECs and HUVECs, we performed RT-qPCR. Contrary to receptor expression, VEGF mRNA levels were higher in HUVECs when compared to iECs (Fig. 3e).

We next tested an alternative differentiation scheme (Supplementary Fig 3c) in which mesodermal differentiation is induced using alpha Minimum Essential Media with no added growth factors or small molecules, followed by EC specification in high VEGF and TGF beta inhibition^28,29^. CD31^+^ iECs purified on day 12 expressed similarly high VEGFR2 mRNA and surface protein levels as cells purified using the Essential 6 Medium containing the WNT agonist CHIR99021 protocol to induce mesodermal differentiation^30^ (Supplementary Fig. 3b, c). Because cells are supplemented with high VEGF (50 ng/mL) during the differentiation and expansion period (Supplementary Fig. 3a), we also differentiated and expanded cells in media containing lower levels of VEGF (10 ng/mL) and found that VEGFR2 levels were unchanged (Supplementary Fig. 3d). It should be noted that the majority of protocols to direct EC maturation from iPSCs include the supplementation of VEGF to induce endothelial fate^31^.

To determine the role of exogenous VEGF during cell expansion, HUVECs were cultured in control basal media (i.e., ECGM), supplemented with VEGF, and media supplemented with both VEGF and the ALK4/5/7 (TGFβ) inhibitor SB431542 for 5 days. In both experimental conditions, VEGFR2 expression decreased significantly compared to control (Fig. 3f). Additionally, in iECs that were expanded in EC Differentiation Media and then cultured in media without VEGF for 5 days, VEGFR2 expression levels remained characteristically elevated (Fig. 3f).

### Inhibition of VEGFR2 causes truncated vascular sprouting and a decrease in glycolysis in iECs

**Fig. 4:**
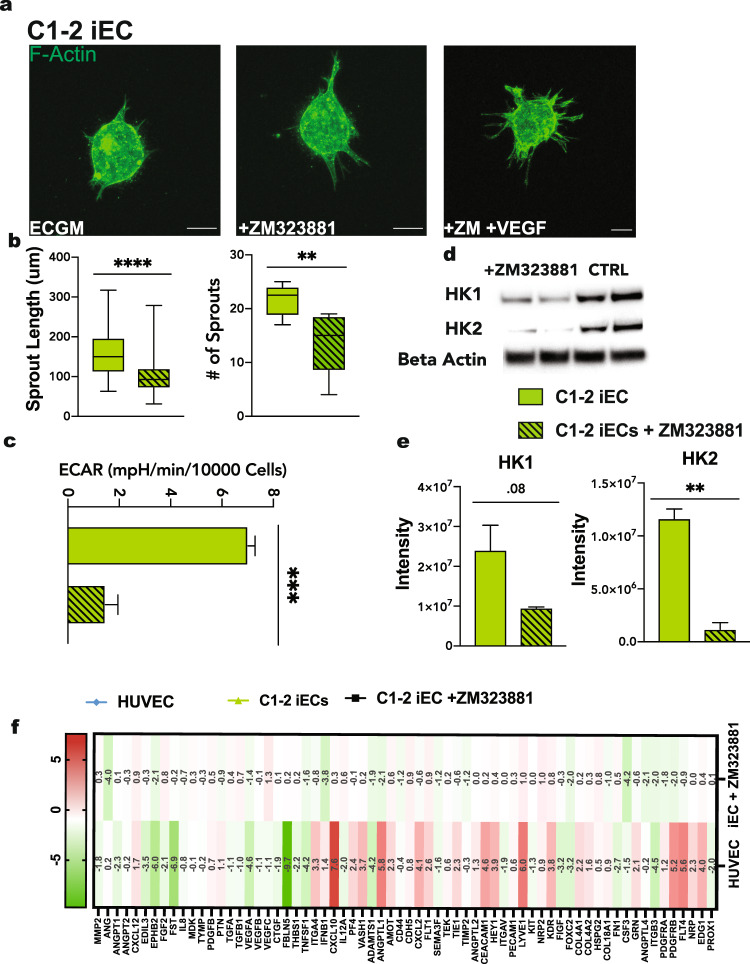
VEGFR2 inhibition in iECs. **a** Representative confocal images of iEC spheroids cultured in ECGM (left), ECGM supplemented with the VEGFR2 inhibitor, ZM323881 (middle), and ZM323881 with VEGF (50 ng/ml; right). **b** Quantification of spheroid sprout number and length in spheroids cultured in culture media supplemented with VEGF (solid), and spheroids cultured in culture media supplemented VEGF and ZM323881 (striped). (*N* = 3, *n* = 15). **c** Extracellular Acidification Rate measured via Seahorse Assay in C1-2 iECs with or without ZM323881 pretreatment (*N* = 2, *n* = 6). **d** Western blot for HK1 and HK2 in hiPSC-ECs cultured in ZM323881 or control conditions and **e** quantification, *N* = 2. **f** Heat map for RT-qPCR results of mRNA expression displayed as ddCT normalized to iECs *N* = 2. Statistical significance levels are set at *p ≤ 0.05, **p ≤ 0.01, ***p ≤ 0.001, and ****p ≤ 0.0001 by two-tailed Student’s t-test and Tukey’s multiple comparison test. For bar graphs: data are presented as mean ± SD. For box and whisker plots: centerline, median; box limits, upper and lower quartiles; whiskers, minimum to maximum value. Scale bar: 100 μm.

To examine whether VEGFR2 activation regulates iEC sprouting, we utilized a 3D spheroids assay where we inhibited VEGFR2 activation using the pharmacological inhibitor ZM323881. Spheroids were generated and embedded in collagen gels as described above and cultured in ECGM supplemented with 1 μM ZM323881, with or without VEGF (50 ng/ml). We found that suppression of VEGFR2 signaling without VEGF resulted in sprouting similar to basal (ECGM) conditions, indicating that ZM323881 treatment had no lethal effects on cells or unintended off-target effects. When spheroids were cultured with both VEGF and ZM323881, the number of sprouts and the length of the sprouts in iEC spheroids were significantly decreased compared to VEGF-only conditions (Fig. 4a, b and Supplementary Fig 4a, b).

To validate the role of VEGFR2 in mediating iEC glycolysis, we cultured cells in ECGM supplemented with ZM323881 for 24 h and analyzed ECAR using a Seahorse assay in glucose media conditions. Results revealed that under VEGFR2 inhibition, glycolytic rates were significantly decreased, with ECAR levels similar to HUVECs (Fig. 4c). As HK1 and HK2 were more highly expressed in iECs, we sought to assess their upstream regulation by VEGFR2. We identified HK1 and HK2 as downstream targets affected by the inhibition of VEGFR2 (Fig. 4d, e). In contrast, when HUVECs were treated with ZM323881, no significant change in HK1 and HK2 was observed (Supplementary Fig 4c, d).

Finally, to deepen our understanding of how VEGFR2 activation regulates iEC phenotype and differences from HUVECs, we used a gene array specific to angiogenesis proteins to compare expression levels between iECs, HUVECs, and iECs under VEGFR2 signaling inhibition. We found that HUVECs have a higher VEGFA, VEGFB, and VEGFC, with a lower expression of VEGFR2, FLT1, and FLT4. HUVECs also had a lower expression of CXC chemokine ligands CXC2, CXCL10, and CXCL12, suggesting an increased migratory phenotype. iECs under VEGFR2 signaling inhibition showed similar expression of most angiogenesis genes analyzed. An increase in expression of VEGF and angiogenin, both pro-angiogenic proteins, indicates a potential compensatory mechanism in iECs attempting to increase angiogenesis under VEGFR inhibition (Fig. 4f).

### VEGFR2 transcription in iECs is regulated by HATs P300 activity

**Fig. 5:**
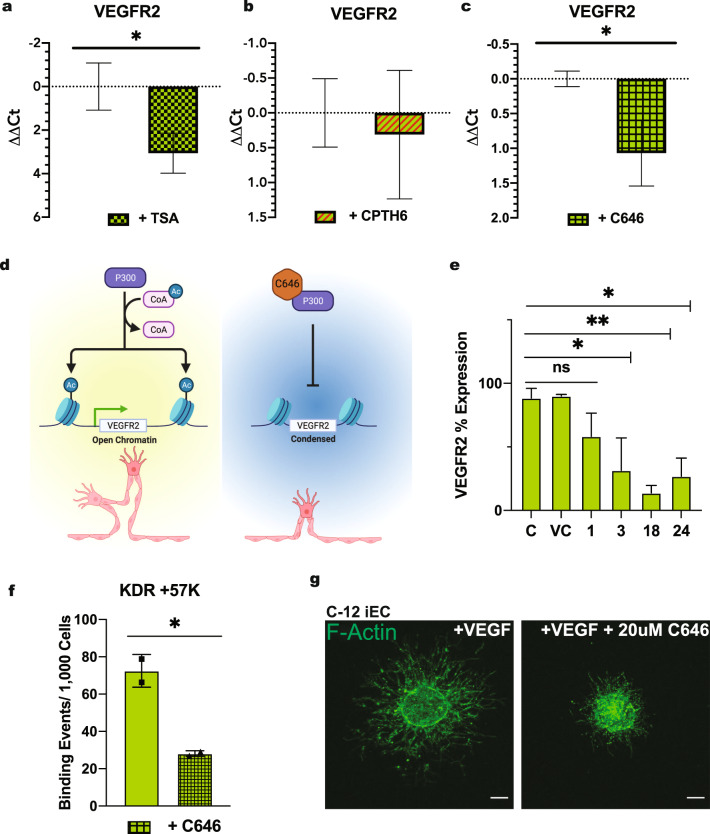
hiPSC-EC VEGFR2 expression is regulated by HAT P300 activity. RT-qPCR for VEGFR2 in C1-2 iECs treated with **a** TSA, **b** CPTH6, and **c** C646. (*N* = 3, *n* = 9). **d** Schematic is outlining of P300 HAT VEGFR2 regulation and C646 inhibition in iECs. Schematic created with BioRender.com. **e** Flow cytometry for VEGFR2 on iECs treated with C646 for varying periods (C-Control, ECGM; VC-Vehicle Control, DMSO; 1,3,18, and 24 h) *N* = 3. **f** ChIP qPCR data are shown as binding events per 1000 cells to the +57 K location at the VEGFR2 transcriptional start site. *N* = 2. **g** Representative confocal microscopy images of C1-2 iECs spheroids cultured in media supplemented with VEGF or VEGF and C646. (*N* = 2, *n* = 10) (*N* = 2, *n* = 10). Statistical significance levels are set at *p ≤ 0.05, **p ≤ 0.01, ***p ≤ 0.001, and ****p ≤ 0.0001 and Tukey’s multiple comparison test by two-tailed Student’s t-test and Tukey’s multiple comparison test. Data are presented as mean ± SD. Scale bar: 100 μm.

Dynamic changes to gene expression in ECs via epigenetic regulation have been explored in cancer biology, as the development of antiangiogenetic drugs as a cancer treatment remains popular^32^. Studies have analyzed the role of epigenetics in normal endothelium, with work identifying DNA methylation^33,34^, HDACs^12–14,35^, and HATs^11,36^ critical to EC function. While DNA methylation has been shown to regulate EC-specific gene expression, bisulfite analysis revealed that the VEGFR2 promoter was not methylated in either EC or non-EC cell types. Additionally, when HUVECs were treated with 5-azacytidine, an inhibitor of DNA methylation, no change in VEGFR2 mRNA was observed^33^. Thus, we set to examine the role of epigenetics in the regulation of VEGFR2 in iECs. We first explored HDACs as potential regulators of VEGFR2 expression in iECs. HDACs remove acetyl groups from histones on DNA, resulting in reduced accessibility of the DNA to transcription factors. Thus inhibition of HDACs is expected to result in increased transcription. However, utilizing Trichostatin A (TSA), a selective inhibitor of class I and II HDACs^37^, we unexpectedly witnessed a downregulation of VEGFR2 expression in iECs (Fig. 5a). Subsequently, we tested HAT activity in iECs. For these studies, we utilized CPTH6 and C646 inhibitors. CPTH6 inhibits the acetyltransferase activity of Gcn5 and pCAF^38^, while C646 inhibits HAT P300^39,40^. After overnight treatment with CPTH6, there was no significant difference in VEGFR2 mRNA levels (Fig. 5b). Conversely, we observed a substantial decrease in VEGFR2 expression in iECs treated with C646 (Fig. 5c).

We next hypothesized that P300 HATs activity could affect sprouting angiogenesis in iECs (Fig. 5d). To further confirm the effect of P300 inhibition on VEGFR2 protein expression, we treated cells for multiple periods and assessed protein expression at 24 h using flow cytometry. A significant decrease in VEGFR2 protein was observed when cells were treated with C646 for 3, 18, and 24 h (Fig. 5e).

Acetylation of histone 3 at lysine 27 (H3K27Ac) is a known marker of active enhancers^41^. Previous work has explored the presence of H3K27Ac at the VEGFR2 promotor site in HUVECs. This study found that when stimulated with high amounts of exogenous VEGF (50 μg/mL) over short periods (0–12 h), HUVECs show increased H3K27Ac bound at multiple promotor sites over time, including VEGFR2^40^. Using ChIP-qPCR, we assessed the binding of H3K27Ac at the promotor region of VEGFR2 in iECs. In both C1-2 iECs and 6.2 iECs, we found a robust H3K27Ac signal at the transcriptional start site of VEGFR2 (Supplementary Fig 5b). Furthermore, iECs treated with C646 resulted in a decrease in H3K27Ac bound to the VEGFR2 TSS region, confirming the direct role of P300 in regulating VEGFR2 transcription and downstream angiogenic activity (Fig. 5f and Supplementary Fig. 5a).

To confirm the functional role of epigenetic regulation on the angiogenic potential of iECs, we assessed sprouting potential under P300 HAT activity inhibition. Spheroids were incubated in media containing VEGF or VEGF with different concentrations of C646 for 24 h. At 24 h, we observed robust sprouting from the control VEGF condition, as expected; however, spheroids incubated with VEGF and C646 displayed reduced sprouting abilities in a dose-dependent manner (Fig. 5g and Supplementary Fig 5b).

### iECs mediated zebrafish caudal fin regeneration is abated by C646 p300 inhibition

**Fig. 6:**
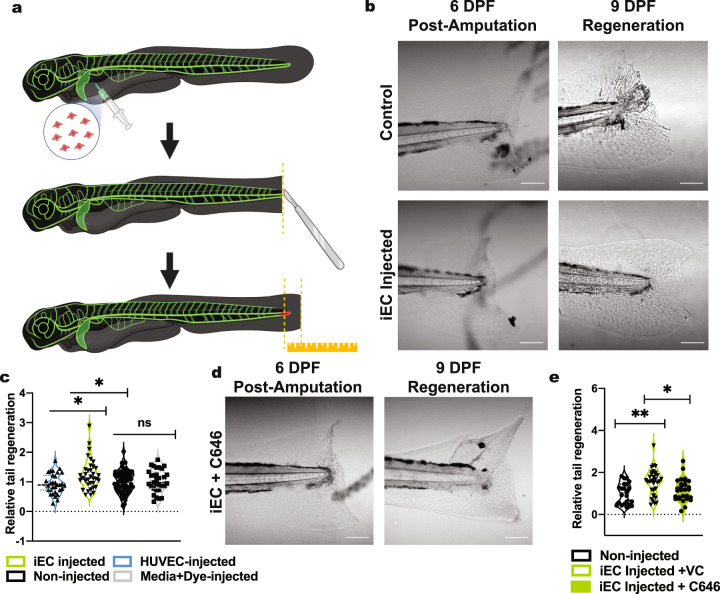
iEC-enhanced zebrafish caudal fin regeneration is abated by p300 inhibition. **a** Schematic outlining zebrafish caudal fin regeneration assay. Schematic created with BioRender.com. **b** Representative images of caudal fin regeneration of control (top) and iEC injected (bottom) at 6 dpf and 9 dpf and **c** quantification including controls (*n* = 35–64). **d** Representative images of C646 iEC injected zebrafish at 6 dpf and 9 dpf and **e** quantification including controls (*n* = 28–33). Statistical significance levels are set at *p ≤ 0.05, **p ≤ 0.01, ***p ≤ 0.001, and ****p ≤ 0.0001 by Tukey’s multiple comparison test. Data are presented as mean ± SD. Scale bar: 100 μm.

We next sought to determine if iECs possess the ability to aid in the regeneration of damaged tissue. We utilized a zebrafish xenograft model as others, and our group has previously shown integration of vascular derivatives from hiPSCs^42,43^. Here, we used a fin amputation model in which the angiogenic response during the first 72 h following injury is critical to rapid regeneration, resulting in the formation of a vascular plexus^44^. Thus, we reasoned that injection of iECs into the fish in conjunction with fin amputation would accelerate regeneration resulting in extensive tissue growth after 72 h compared to untreated fish. Intra-pericardial injection of C1-2 iECs or control media at 6 days post fertilization (dpf) was followed by caudal fin amputation (Fig. 6a). At 9 DPF, fish injected with iECs showed increased caudal fin regeneration than non-injected fish, media-injected control fish, and HUVEC injected fish (Fig. 6b, c). We next injected iECs treated with C646 to determine the role of VEGR2 expression in iEC-aided tail regeneration. We found that injection of C646-treated iECs reduced caudal fin regeneration compared to non-treated cells (Fig. 6d, e).

## Discussion

Since their advent, stem cell-derived vascular cells have been touted as a potential cell-based therapy for mediating diseased endothelium. Our lab and others have shown these cells’ in vivo regenerative capabilities in several murine models^45–49^. While recent studies have presented functional differences between iECs and mature ECs, no study has proposed a mechanism behind the observed differences. Here, we reveal HATs P300 as a critical regulator of the angiogenic potential of iECs via regulation of VEGFR2 transcription.

Our group investigated the use of iECs and primary human retina ECs (HRECs) as potential retinopathy therapeutics in previously published work. Key findings of this work show increased network formation in vitro and in vivo of iECs compared to primary HRECs^50^. iEC functionality has also been extensively compared against human microvascular ECs (HMVECs) and HUVECs^51–54^. We chose to use HUVECs as controls for the work presented here as they are considered the “gold standard” for analyzing mechanisms underlying angiogenesis and tip-cell phenotype in vitro. We sought to examine sprouting angiogenesis using a spheroid assay to observe the analogous differences between HUVECs and iECs in 3D hydrogels, a widely used method to explore how ECs invade their environment to promote vessel growth in response to different stimuli^15^. Without VEGF supplementation, iECs outperform HUVECs, forming more and longer sprouts over 24 h. Unexpectedly, we found that HUVECs appeared to be migrating away from the spheroids instead of sprouting outward. This phenotype persisted even when cells were supplemented with VEGF.

The Carmeliet group has published extensively on the crucial role glycolysis plays in the phenotype of ECs and, more specifically, sprouting tip cells. They showed over several works that glycolysis plays a vital role in the organization of the tip cell at the leading edge of angiogenesis and promotes filopodia formation^3,55,56^. To analyze glycolysis in iECs, we utilized a Seahorse Assay, which measures lactate accumulation, a byproduct of glycolysis, as the ECAR. This assay provides a real-time analysis of glycolytic rate in cells under various chemically controlled environments. In addition, we directly measured the expression of glycolysis enzymes in cells via Western Blotting. While HUVECs expressed significantly more protein phosphofructokinase (PFKP), an enzyme that catalyzes an irreversible essential step in glycolysis, we see increased HK1/HK2 and PFKFB3 expression in iECs, both being key in controlling glycolytic flux^57^. Ideally, the analysis should include measurements of the glycolytic rate as cells undergo sprouting angiogenesis in 3D environments. However, this analysis though is currently not possible with the Seahorse Assay platform, and is complicated by using thick hydrogels to embed spheroids.

Cell migration is a highly coordinated process requiring a specific cytoskeleton reorganization to propel the cell forward. The cell’s leading edge protrudes in the direction of motion via actin polymerization. Integrins at the leading edge interact with the extracellular matrix to anchor the protruded cell body, then translocation of the cell body, followed by tail retraction. Filopodia and lamellipodia are actin projections formed at the leading edge of the cell. While lamellipodia are found in most migratory cells^58^, filopodia are involved in several cellular processes in addition to migration^59^. A key role of filopodia is to probe the extracellular space. As such, filopodia have been shown to contain receptors along their shafts that interact with mitogens in the environment. For instance, endothelial tip cells form filopodia to facilitate migration toward VEGF gradients^5^. To measure filopodia, we employed a similar method to the previous works^3^ by seeding cells sparsely on 2D surfaces and using image quantification to measure filopodia number. While this method allows for clear visualization of filopodia, this assay lacks many physiological variables that contribute to the activated tip cell phenotype. To overcome this, we used a microfluidic assay to image and quantify cells directly responding to and migrating towards a VEGF gradient. Although stress fibers could not be directly imaged at high magnifications using the microfluidic device, we can assess cellular motility in real-time. The behavior of HUVECs in the device complemented the migratory behavior we observed in HUVEC spheroids. In media with and without VEGF, HUVECs were highly motile. This behavior indicates a lack of response to VEGF and an overall random migratory phenotype. In contrast, iECs only migrated in response to a VEGF gradient in the device, showing a distinct phenotype as VEGF-responsive ECs.

Next, we hypothesized that a differential expression of VEGF receptors caused the divergence in VEGF responsiveness. Membrane-bound VEGFR2 serves as the primary receptor for VEGF-A and is a tyrosine kinase receptor transcribed by the KDR gene^60^. Indeed, VEGFR2 was expressed higher at the mRNA and protein levels in iECs. Importantly, we also measured for the first time increased phosphorylation of VEGFR2 in iECs. All experiments measuring VEGFR2 expression were conducted in non-VEGF supplemented conditions; this suggests that iECs VEGFR2 activation is not dependent upon exogenous VEGF to activate VEGFR2 downstream targets. We next sought to understand the role VEGFR2 played in the angiogenic phenotype we observed in iECs. To do this, we inhibited VEGFR2 utilizing ZM323881, a pharmacological inhibitor of VEGFR2 phosphorylation. We observed a significant decrease in iEC sprouting and glycolysis in both conditions, while HUVECs under VEGFR2 inhibition displayed no measurable differences in any assay. It is important to note that this is evidence showing that VEGFR2 inhibition modulates HK1 and HK2 activity.

Several recent studies have revealed a link between epigenetics, aging, and cellular regeneration^61^. Epigenetics in iPSCs and their derivatives have also been studied. Hu et al. explored how differential cell sources for iPSCs affected EC differentiation, demonstrating how epigenetic memory in iPSCs can affect EC phenotype^62^. Furthermore, several studies have noted the effects of modulating the epigenome on the function of ECs, including truncated sprouting, migration, and network formation on 2D surfaces^9,11–14,36^. We have found explicitly that HDAC6 modulates cilia formation in iECs, thus impacting their response to shear stress^63^. Nonetheless, we saw no differential expression of VEGFR2 in iECs or HUVECs when methylation was inhibited.

P300 is an active enzyme in cells that functions as HATs and a co-activator of transcription factors, including HIF-1alpha^64^. We specifically inhibited HATs activity using the small molecule C646. We observed downregulation of VEGFR2 mRNA and protein in the presence of C646. Additional analysis using a spheroid sprouting assay revealed a direct link between P300 HAT activity and vascular sprouting. While we used ChIP to assess the binding of the open chromatin signature, H3K27ac, this assay could be used to look at the binding of transcription factors to the VEGFR2 promoter site. Additional small molecule inhibitors and other epigenetic signatures can be analyzed in future studies.

Zebrafish have emerged as a leading model organism in biomedical research. Particularly, genetics between vascular development are well conserved between humans and zebrafish^65^, making the zebrafish an excellent model to study the angiogenic potential of human stem cells. Additionally, zebrafish possess a remarkable ability to regenerate damaged appendages and thus have long been used as a model to study vertebrate organ restoration. The larval caudal fin, in particular, is easily manipulated and quickly regenerates, making it an attractive assay to test various manipulations^66^. After an injury, blastema formation precedes cell proliferation and rapid outgrowth. Several signaling pathways regulate this process, including Sonic hedgehog, Wnt/β-catenin, Retinoic Acid, IGF, and Notch. For example, tail regeneration can be inhibited by treatment with cyclopamine (hedgehog antagonist) and dorsomorphin (BMP inhibitor). Epigenetic regulation, including transient DNA demethylation soon after injury, is another known mediator of tail regeneration^67^. Multiple types of human ECs have been injected in developing zebrafish larvae previously in efforts to develop vascular disease models. Importantly, injected ECs showed stable integration into the vasculature and caused no developmental abnormalities^68^. We designed a xenograft experiment to compare iECs and HUVECs impact on tail regeneration in larval fish. iECs were consistently capable of enhancing the kinetics of tail regeneration compared to non-injected and HUVEC-injected controls. Inhibition of P300 HAT activity impaired the ability of iECs to promote tail regeneration, validating this mechanism as a critical mediator driving angiogenic potential.

In conclusion, this study reports an epigenetic regulation of iEC angiogenesis and regenerative capacity. Our approach utilized in vitro platforms to determine iEC angiogenic capacity in a comparative study with HUVECs. While previous studies have demonstrated the ability of iECs to regenerate vascular tissue in vivo, to date, no regulation mechanism has been proposed. Furthermore, we validated our findings that iEC angiogenesis can be modulated by P300 inhibition in vitro and in vivo using the zebrafish caudal fin regeneration model. Our results highlight the ability to regulate the regeneration of iECs and offer further insight into the role of epigenetics in controlling the regenerative capacity of these cells. Overall, this data reveal increased VEGFR2 expression in iECs regulated by innate epigenetic modification.

## Methods

### Cell culture

Human iPSC lines (Supplementary Table 1) were maintained on human recombinant Vitronectin (ThermoFisher Scientific, Waltham, Massachusetts) protein-coated plates and supplemented with Essential 8 media (ThermoFisher Scientific). Cells were seeded at 25% confluence and expanded over 4 days before passage, with daily media changes. HUVECs (Promocell, Heilberg, Germany) were purchased from the vendor at passage one and used through passage five. HUVECs were cultured in Endothelial Cell Growth Media (ECGM) plus supplement with media changed every 48 h. Ethics committee approval was obtained from the respective institutional ethics committee for each human cell line, and all patients provided written informed consent for the use of their cells for future research, specific details are provided in Supplementary Table 1.

### hiPSC differentiation to ECs

iECs differentiation was induced as previously^42,63,69,70^, where hiPSCs cultured to 60–80% confluency with Essential 6 medium (ThermoFisher Scientific) supplemented with 6 μM CHIR (STEMCELL Technologies, Vancouver, Canada), with media changed daily over 48 h. After 48 h, cells were digested in TrypLE Express and seeded on Collagen I coated plates at 2 × 10^4^ cells/cm^2^ in Endothelial Cell Differentiation Media containing: ECGM (Promocell) supplemented with 10 μM SB-431542 (Cayman Chemical Company, Ann Arbor, MI) and 50 ng/mL VEGF (R&D Systems Inc, Minneapolis, MN), with additional supplementation in 10 μM Y-27632 for the first 24 h. After the first 2 h, the media was changed every other day for an additional six days. EC specification was also examined in 10 ng/mL VEGF (R&D Systems Inc). Differentiation scheme in which mesoderm was induced using alpha Minimum Essential Media (ThermoFisher Scientific) followed by EC specification in high VEGF (R&D Systems) and SB-431542 (Cayman Chemical Company) was performed as previously^28,29^.

### Isolation and expansion of iECs

CD31 expressing cells were isolated on day 8 of differentiation via magnetically activated cell sorting (MACS; Miltenyi Biotec Bergisch Gladbach, Germany) following the manufacturer’s protocol. After washing with 1× phosphate-buffered saline (PBS, ThermoFisher Scientific), cells were harvested with TrypLE Express and resuspended in MACS buffer (0.5 EDTA and 0.5% BSA in PBS). Cells were then incubated with 10 μl of PE-conjugated anti-human CD31 (BD Biosciences, San Jose, CA) for 10 min at 4 °C. After incubation, the unbound primary antibody was removed by washing with MACS buffer twice. Next, 20 μl of anti-PE microbeads (Miltenyi Biotec Bergisch Gladbach, Germany) were added to 80 μl of cells suspended in MACS buffer and incubated for an additional 15 min at 4 °C. Cells were washed with MACS buffer and separated using the MS MACS separation column (Miltenyi Biotec). Following separation, CD31 and VECAD enrichment were confirmed using flow cytometry as previously^71^ and detailed below in a separate section. Finally, CD31+ cells were seeded on type I collagen-coated plates and maintained in EC differentiation media. For all experiments, media was switched to ECGM without growth factor supplementation for 24 h unless otherwise noted. All experiments used iECs between passages 1 and 3.

### Spheroid assay

Cells were grown to confluence then detached using TrypLE. Cells were counted and resuspended at a density of 50,000 cells/mL in ECGM containing 20% methylcellulose. Hanging drops were formed by pipetting 20 μL droplets onto the top of a petri dish and inverting. Droplets were incubated with PBS overnight. After 24 h, spheroids were collected and embedded in type I collagen gels as follows:^72^ to prepare 1 mL of collagen gel solution, 800,000 cells were resuspended in 400 μL of Medium 199(1X), 40 μL of Medium 199(10X), and 350 μL of 7.1 mg/mL Rat Tail Collagen I. The pH of the solution was adjusted by titrating 1 M NaOH up to 10 μL. 56 μL of the mixture was added to the wells of a 96 well plate. Gels were polymerized at 37 °C for 30 min. ECGM supplemented with 50 ng/mL of VEGF was added after 30 min.

### Seahorse assay

Glycolysis Stress Test Seahorse-based assay was done according to the manufacturer’s protocol. Cells were seeded in ECGM media at a density of 60,000 cells per well in an XF24 well plate (Agilent, Santa Clara, CA) coated with Collagen I. 1 h before Seahorse analysis media was switched to Seahorse XF DMEM (Agilent), containing no phenol red, bicarbonate, glucose, pyruvate, or glutamine, and incubated in a non-CO_2_ incubator. The Seahorse XF24 Analyzer (Agilent) was used to measure extracellular acidification (ECAR) and oxygen consumption rate (OCR) in real-time. Non-glycolytic acidification was measured first in the absence of both glucose and pyruvate. Final concentrations of 10 mM glucose (Sigma Aldrich, St. Louis, MO), 1 µM oligomycin (Sigma Aldrich), and 5 mM 2-deoxy-D-glucose were added to establish cellular glycolysis, glycolytic max, and glycolytic reserve, respectively. Cells were stained for DAPI, and cell number was quantified via Imaris. Values were normalized to the total cell number per well.

### Inhibition studies

For all inhibition studies, cells in each condition were cultured in ECGM for 24 h. After 24 h, the inhibitor was diluted in DMSO and added to the culture media, including CPTH6 (Cayman Chemical Company) at 30 μM^73^, Trichostatin A- 1 μM (Sigma Aldrich) at 1 μM^13,74^, ZM323881 (Selleckchem) at 1 μM^75^ and C646 (EMD Millipore) at 10–30 μM^40^. Vehicle alone served as control.

### Immunofluorescent staining and imaging

Cells were cultured on coverslips or as spheroids and fixed in 4% formaldehyde. Formaldehyde was removed, then the samples were washed with 1× PBS. Cells were then permeabilized with 0.1% Triton X-100 (Sigma-Aldrich) for 10 min. Samples were washed then incubated with 1% BSA solution for one hour at room temperature. Following blocking, samples were washed with PBS and incubated with Phalloidin overnight at 4 °C. Samples were then washed three times with 1× PBS, incubated for 3 min with DAPI (Roche Diagnostics), washed, and placed onto glass slides using mounting media (Dako, Carpinteria, CA). Cells were imaged using a Zeiss Laser Scanning Microscope 780 confocal microscope. Cell filopodia were quantified using the Image J plug-in FiloQuant.

For the spheroid assay, after 24 h, collagen gels with spheroids were fixed in 2% formaldehyde for 20 min. Formaldehyde was removed, and gels were washed three times in 1× PBS. Gels were then incubated in 1% Triton-X 100 (Sigma Aldrich) for 10 min, then rinsed in 1× PBS in 30 min intervals. Following, gels were blocked in 10% bovine serum albumin (BSA) solution for 1 h at room temperature and incubated with a conjugated phalloidin probe for 2 h at room temperature. Gels were rinsed with a 0.05% TWEEN 20 (Sigma Aldrich) solution and stored in unsupplemented 1× PBS until imaged. Spheroids were imaged using an LSM 780 or 800 confocal microscope. The number of sprouts per spheroid and the average sprout length were quantified using the Filament package in the ImarisSoftware (Bitplane).

### Flow cytometry

Cells were harvested for analysis using TrypLE (Invitrogen) disassociation buffer and collected in 100 μL of 0.1% bovine serum albumin. After collection, cells were incubated with conjugated antibodies against proteins of interest for 30 min on ice. Cells were washed three times with 0.1% BSA and passed through a 40 μm cell strainer. Flow analysis was conducted on a BD FACSCaliber flow cytometer. Following the manufacturer’s instructions, dead cell populations were gated out with forward-side scatterplots. All analyses were conducted using IgG-PE or IgG-FITC (BD) isotype controls. All analyses were performed using FCS Express 6 Flow (De Novo Software, Pasadena, CA). The gating strategy is outlined in Supplementary Fig 6.

### Western blotting

Cells were lysed using RIPA Buffer (Thermo Fisher) with 1X Protease and Phosphatase Inhibitor Cocktail (Thermo Fisher Scientific). Protein was quantified using the BCA Assay (Thermo Fisher Scientific). 20–25 μg of protein from each sample was boiled at 95 °C for 5 min, then loaded into a 4–12% Bis-Tris Protein Gel (Thermo Fisher Scientific). Proteins were transferred to a PVDF membrane (BioRad, Hercules, CA) via wet transfer in a Bio-Rad Criterion system for 60 min. Total protein was quantified using a Ponceau-S stain and imaged using the ChemiDoc XRS + System (BioRad). Membranes were blocked in 5% milk for 1 h then incubated in primary antibody (see Supplementary Table 2) overnight with gentle agitation. Membranes were washed 3× for 10 min in Tris-buffered saline with 0.1% Tween-20 (Sigma Aldrich, St. Louis, MO) (TBST), then incubated for one hour with anti-rabbit IgG, HRP-linked antibody (Cell Signaling Technologies Danvers, MA) with gentle agitation. The membrane was washed 3× with TBST to visualize the protein of interest substrate (Thermo Fisher Scientific) was added, and membranes were imaged using the ChemiDoc XRS + System (BioRad). Blots were analyzed using Image Lab software (BioRad); bands were normalized to endogenous control protein levels. Blots to be used for multiple analysis were stripped in stripping buffer (containing glycine, SDS, and Tween-20) by washing 3× with buffer, 2× with PBS, 2× with TBST, blocked in 5% milk, then substrate was added to ensure complete removal of antibody. All blots derive from the same experiment and were processed in parallel.

### Quantitative reverse transcription polymerase chain reaction (qRT-PCR) gene expression analysis

Total RNA was extracted using TRIzol reagent (Thermo Fisher Scientific, Waltham, Massachusetts) and purified using the Direct-zol RNA Miniprep Kit (Zymo Research, Irvine, CA). RNA quality was assessed using a nanodrop spectrophotometer. Complementary DNA (cDNA) was generated using Moloney murine leukemia virus reverse transcriptase and oligo(dT) primers (Promega, Madison, WI) per the manufacturer’s protocol. The TaqMan Universal PCR Master Mix and Gene Expression Assays were used for genes of interest. TaqMan PCR was performed using the QuantStudio 5 PCR System. The comparative computerized tomography method was used to calculate the amplification difference between samples as normalized to the endogenous control gene TBP. For RT qPCR array experiments, RNA was isolated as above. cDNA was generated using the GoScript Reverse Transcriptase Kit (Promega). The TaqMan Fast Advanced Master Mix (Thermofisher Scientific) was used as recommended by the manufacturer. The TaqMan Array Human Angiogenesis 96 well plate was used.

### Microfluidic assay

Microfluidic channels (10 µm W, 10 µm H, 200 µm L) were fabricated as previously described. Briefly, molds were made via photolithography. SU‐8 3010 negative photoresist (Microchem, Newton, MA, USA) was spun to a thickness of 10 μm on a mechanical grade silicon wafer (Wafer World, West Palm Beach, FL), soft-baked, and exposed through a mask defining microchannels on an EVG620 mask aligner (EVG, St. Florian am Inn, Austria). After a postexposure bake, the photoresist was developed with a SU‐8 developer, and the patterned wafer was rinsed with isopropanol. A layer of SU‐8 3025 was spun on top of the microchannels to a thickness of 50 μm soft-backed and exposed through a mask defining the cell‐and medium‐containing channels of the device. The new layer of photoresist was baked postexposure and developed. The completed wafer was hard-baked for 10 min at 150 °C and treated overnight with vapor phase (tridecafluoro‐1,1,2,2,‐tetrahydrooctyl)‐1‐trichlorosilane (Pfaltz & Bauer, Waterbury, CT, USA). The final microfluidic devices were formed by using replica molding with polydimethylsiloxane (PDMS; Sylgard 184 kit; Dow Corning, Midland, MI, USA). PDMS prepolymer and cross‐linker were mixed at a 10:1 ratio, poured over the wafer, degassed, and cured at 85 °C for 2 h. PDMS devices were peeled from the wafer mold and diced.

Cells were resuspended in ECGM free of VEGF A at a 5 × 10^6^/mL density. Cell suspension (20–40 μL) was seeded into microfluidic devices via pressure-driven flow. Cells were maintained at 37 °C, 5% CO_2_, and allowed to adhere to the device for 15–20 min before introducing the VEGF gradient. For devices containing a VEGF gradient, the bottom three wells were filled with ECGM free of VEGF A, while the top well was filled with ECGM containing VEGF A. For devices without a gradient, all media inlet wells were filled with ECGM free of VEGF A. Cells were imaged every 10 min for 12 h on an inverted Nikon Eclipse Ti Microscope. Cells were maintained on a temperature and CO_2_-controlled stage top incubator (Okolab, Pozzuoli, Italy; Tokai Hit, Shizuoka-hen, Japan) during these experiments. Cell migration was tracked as previously described using ImageJ^76^. Percent cell entry was calculated based on the fraction of cells within 100 µm of the channel entrance that entered confining microchannels during the duration of the experiment.

### Zebrafish maintenance and husbandry

Animal studies described herein were performed per the National Institutes of Health (NIH) Office of Laboratory Animal Welfare (OLAW) policies regarding studies conducted in vertebrate species. Animal protocols were approved by the Animal Care and Use Committee of the Johns Hopkins University School of Medicine. Zebrafish were maintained using established temperature and light cycle conditions (28.5 °C, 14 h of light/10 h of dark). For the experiments described below, we used a transgenic line *Tg(VEGFR2:GRCFP)* that labels the zebrafish vasculature with GFP^77^.

### Zebrafish caudal fin regeneration assay

In vivo experiments in fish began at 6 days post fertilization (dpf) following GFP screening and placement of larvae placement in a 32 °C incubator at 5 dpf. 32 °C was chosen as a temperature to allow injected cells to better survive in the fish without deleterious effects on fish survival. For all caudal fin amputation assays, groups of ~8–12 larvae per condition were anesthetized in 0.016% tricaine and mounted laterally in 1% low melting agarose gel (maintained at 42 °C before use). After allowing agarose to set, larvae were overlayed with E3/PTU media + 0.016% tricaine. For injection of iECs, HUVECs, and media+dye controls, larvae were placed under a PLI-100 picospritzer (Harvard Apparatus) and injected with 10 nL of solution. For both iEC and HUVEC injections, cell solutions contained 40k cells per µL. Immediately following injection, tail fins were amputated using a Pharmacia Superblade and then imaged with an Olympus Fluoview FV1000 confocal microscope (20× water immersion objective, 0.95NA) with a focus on the caudal fin. Larvae were then placed in E3/PTU media individually in a 24-well plate. 3 days later, at 9 dpf, larvae were remounted in agarose and imaged on the confocal microscope to assess tail regeneration. To analyze tail regeneration kinetics, images for each fish at the post-amputation (6 dpf) and post-regeneration (9 dpf) stage were loaded into Fiji/ImageJ, and the distance from the end of the caudal vein to the end of the fin was measured by drawing a straight segmented line and using Analyze-Measure to give two values for each fish. The difference between the 9 and 6 dpf tail length was then measured for each fish in each condition and normalized to the average length of regeneration in control non-injected fish. For each condition including, iECs, HUVECs, Media+Dye, and iECs + P300 inhibitor C646 (1 h treatment), at least 3 experimental replicates were performed with a group of non-injected control larvae used across all assays.

### Graphs and statistics

We performed statistical analysis using GraphPad Prism 8 (GraphPad Software Inc.). Biological replicates are indicated as *N* and technical replicates are indicated as *n*, and are detailed in figure legends. We also used this software to perform a two-tailed Student’s t-test and Tukey’s multiple comparison test to determine significance. Replicates are indicated throughout the figure captions. All graphical data are reported as means ± SD. Non-significant differences were labeled in figures as “ns” while significant differences were reported *P ≤ 0.05, **P ≤ 0.01, ***P ≤ 0.001, and ****P ≤ 0.0001.

### Reporting summary

Further information on research design is available in the Nature Research Reporting Summary linked to this article.

## Supplementary information

Supplementary Video 1

Supplementary Video 2

Supplementary Information

REPORTING SUMMARY

## Data Availability

The data that support the findings of this study are available from the corresponding author upon reasonable request.
