# Supplementary material for: Intrinsic epigenetic control of angiogenesis in induced pluripotent stem cell-derived endothelium regulates vascular regeneration
Source: NPJ Regen Med. 2022 May 12;7:28. doi: 10.1038/s41536-022-00223-w (PMC9098630; doi:10.1038/s41536-022-00223-w)
Supplement: Supplementary file 3 — Supplementary Information [file 41536_2022_223_MOESM3_ESM.docx]

**Intrinsic epigenetic control of angiogenesis in induced pluripotent stem cell-derived endothelium regulates vascular regeneration**

Bria L. Macklin^1^, Ying-Yu Lin^1^, Kevin Emmerich^2^, Emily Wisniewski^1^, Brian M. Polster^3^, Konstantinos Konstantopoulos^1^, Jeff S. Mumm^2^, Sharon Gerecht^1,4,5,6,7*^

**Supplementary Figures**

**Supplementary Figure 1**


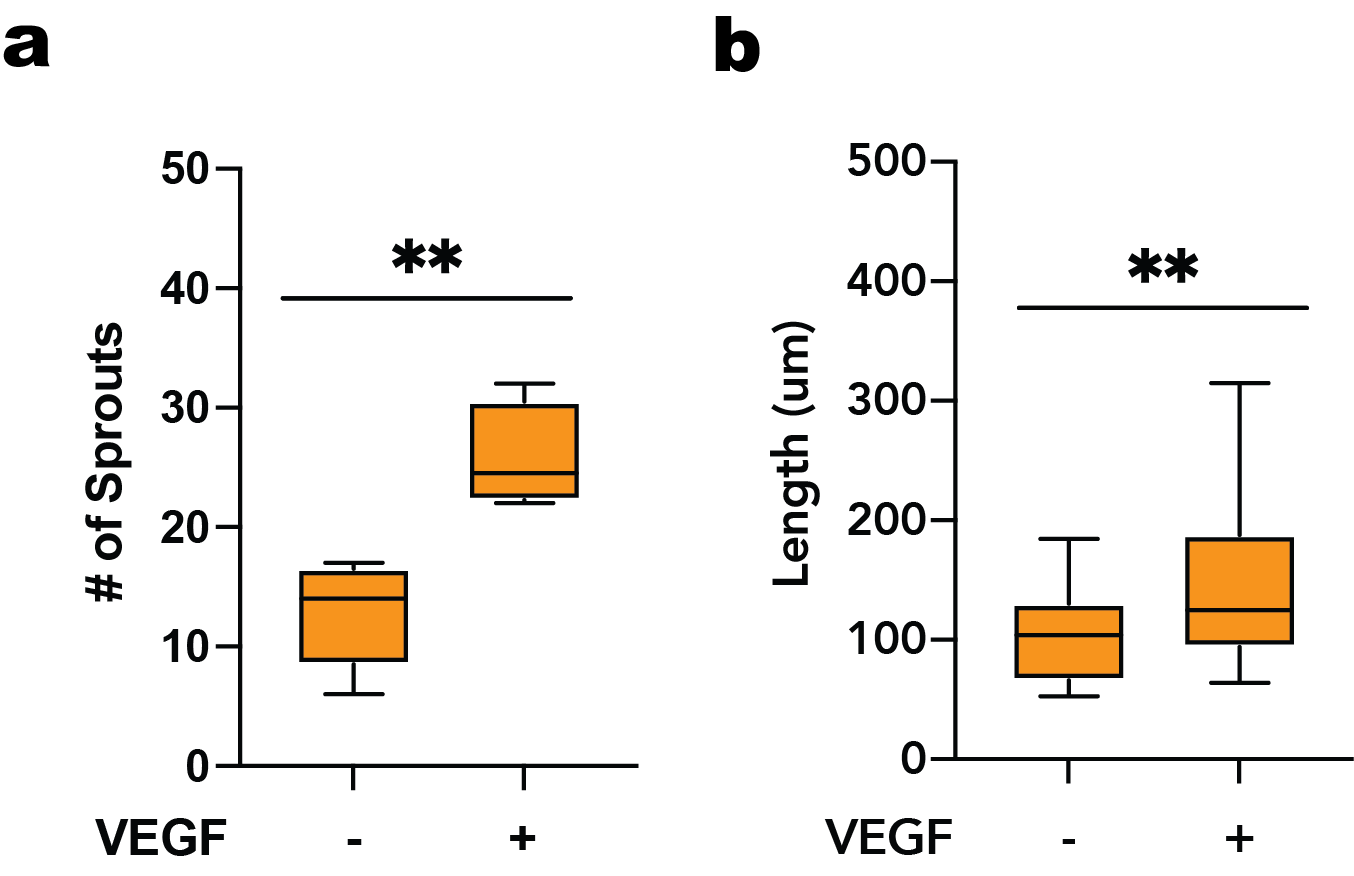


**Supplementary Figure 1. Sprouting in 6.2 iECs.** Sprouting quantification of iECs derived from the 6.2 line including (a) # of sprouts and (b) sprout length in media supplemented with or without VEGF. (N=3, n=25). Statistical significance levels are set at **p* ≤ 0.05, ***p* ≤ 0.01, ****p* ≤ 0.001, and *****p* ≤ 0.0001 by 2-tailed Student’s t-test and Tukey’s multiple comparison test. Data are presented as mean ± SD.

**Supplementary Figure 2**
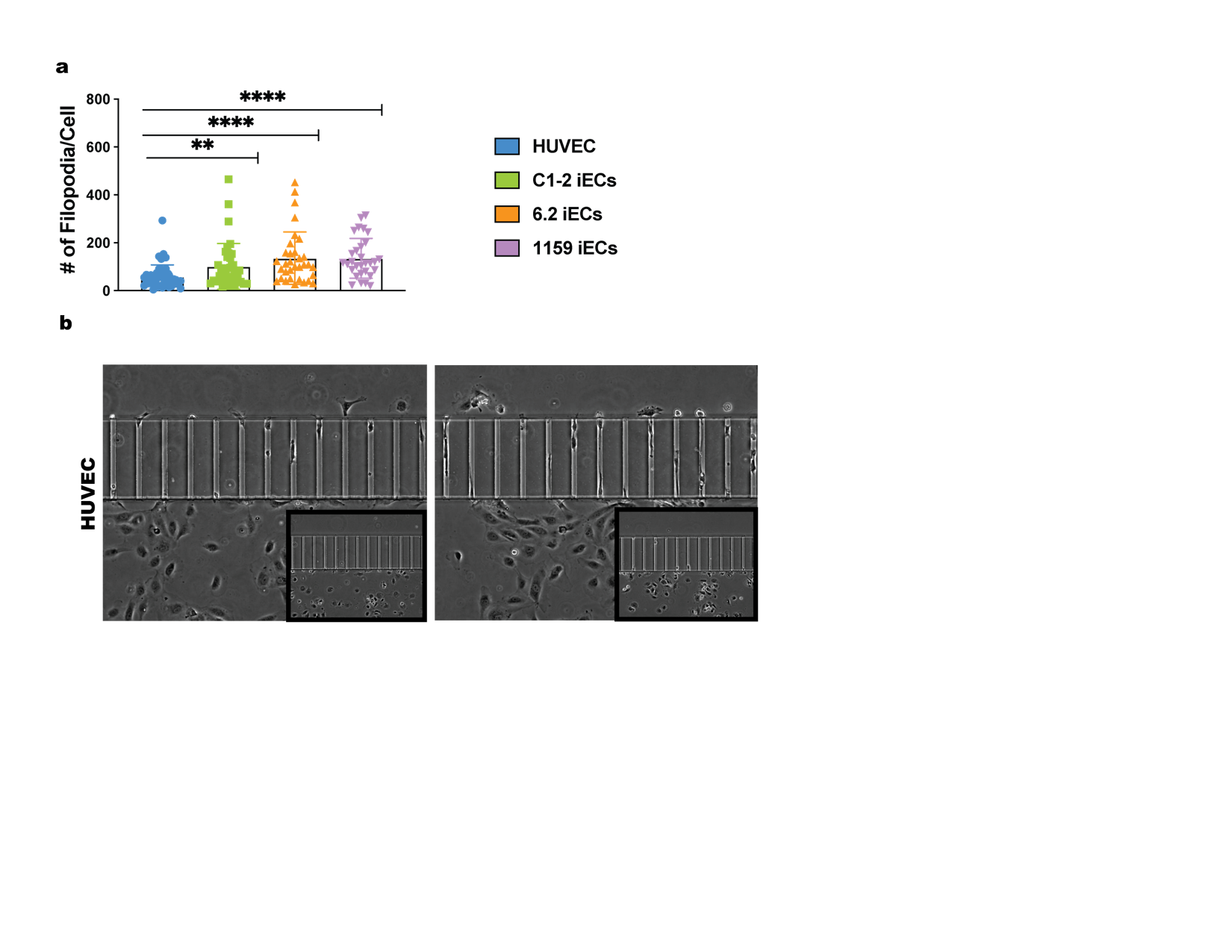


**Supplementary Figure 2**. **Filopodia in iECs from several lines and** **HUVECs Migration** (a) Quantification of filopodia via FiloQuant in iECs from the different hiPSC lines. 15 cells in each experiment with N=3. Graph plotted with outliers. (N=3, n=30-45) (b) image of HUVEC migration with (right) and without (left) VEGF gradient at t=6 hours. The boxed images show cells at t=0. Statistical significance levels are set at **p* ≤ 0.05, ***p* ≤ 0.01, ****p* ≤ 0.001, and *****p* ≤ 0.0001 by Tukey’s multiple comparison test. Data are presented as mean ± SD.

**Supplementary Figure 3**

**Supplementary Figure 3. Validation of VEGFR2 expression.** (a) RT-qPCR results are relative to HUVECs for VEGFR2 mRNA expression of hiPSC-ECs from different cell lines and differentiation strategies, N=2. (b) Flow cytometry results for VEGFR2 protein expression, N=2. (c) Differentiation strategies used, E6+CHIR (top) differentiation and alpha MEM (bottom) differentiation. MD, mesodermal induction; ES, endothelial specification. (d) VEGFR2 expression of C1-2 hiPSC-ECs differentiated and expanded in low VEGF (10 ng/mL). N=2 Statistical significance levels are set at **p* ≤ 0.05, ***p* ≤ 0.01, ****p* ≤ 0.001, and *****p* ≤ 0.0001 by 2-tailed Student’s t-test and Tukey’s multiple comparison test. Data are presented as mean ± SD.

**Supplementary Figure 4
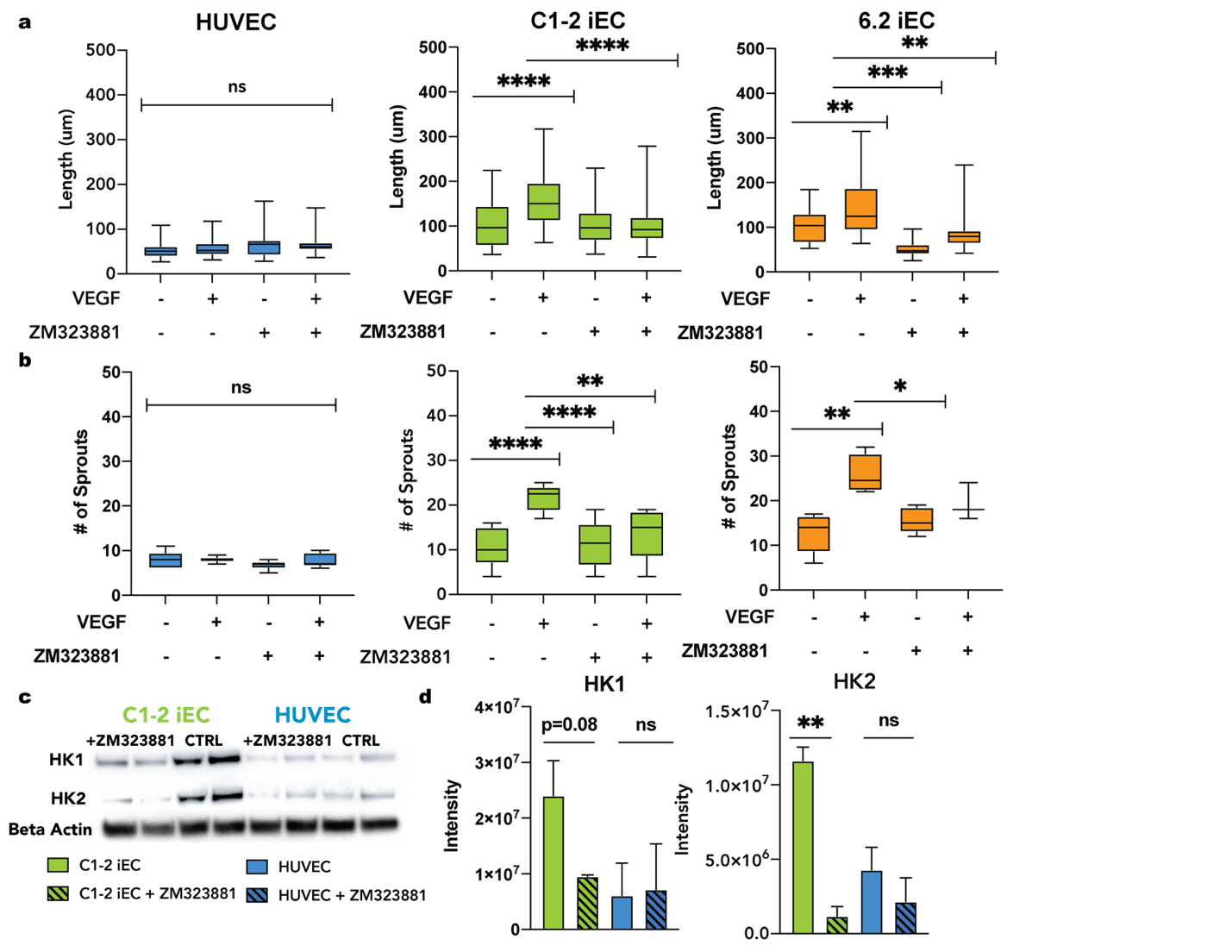
**

**Supplementary Figure 4. VEGFR2 inhibition in HUVECs.** Summary of sprouting quantification including (a) sprout length and (b) # of sprouts in HUVECs, C-12 iECs, and 6.2 iECs with and without VEGF and inhibitor ZM323881. Note that controls with no ZM323881 are data presented in Fig 1b,c. Experiments were performed with all 4 conditions presented here. (N=3, n=25-30) (c) Western blot for HK1 and HK2 in HUVECs and iECs with and without VEGFR2 inhibition and (d) quantification, N=2. Statistical significance levels are set at **p* ≤ 0.05, ***p* ≤ 0.01, ****p* ≤ 0.001, and *****p* ≤ 0.0001 by 2-tailed Student’s t-test and Tukey’s multiple comparison test. Data are presented as mean ± SD. Scale bar: 100 μm.

**Supplementary Figure 5**

**Supplementary Figure 5. CHiP analysis and dose-response to P300 inhibition.** (a) Same ChIP qPCR data from Fig 1f compared to iECs derived from the 6.2 line are shown as binding events per 1000 cells to the +57K location at the VEGFR2 transcriptional start site. N=2(b) Representative confocal microscopy images of C1-2 iEC spheroids in collagen gels in culture media supplemented with VEGF or VEGF and varying concentrations of C646. Statistical significance levels are set at **p* ≤ 0.05, ***p* ≤ 0.01, ****p* ≤ 0.001, and *****p* ≤ 0.0001 and Tukey's multiple comparison test by 2-tailed Student's t-test and Tukey's multiple comparison test. Data are presented as mean ± SD. Scale bar: 100 μm.


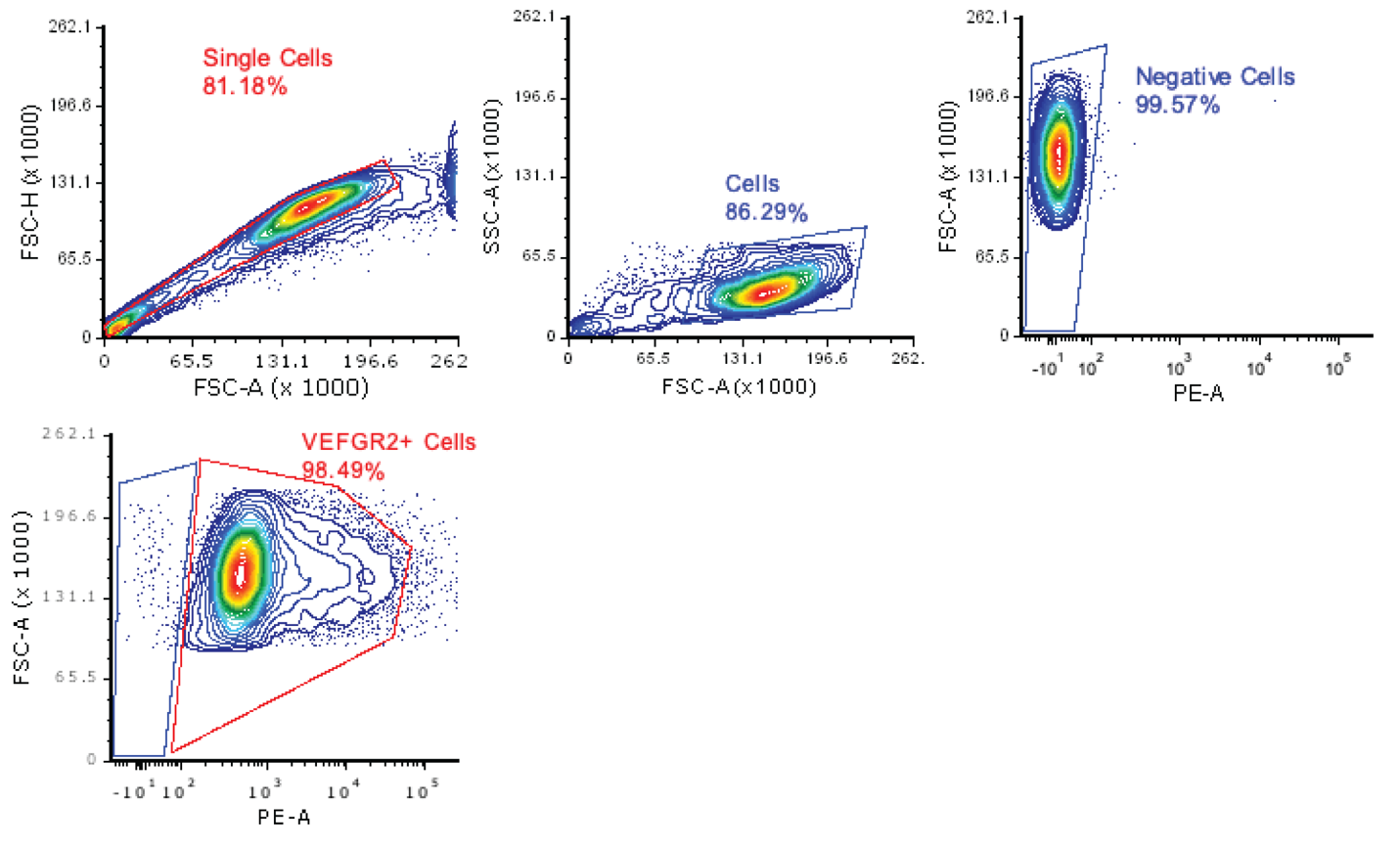


**Supplementary Figure 6. Gating strategy of flow cytometry analysis.** Events were gated for single events, then live cells, followed by gating for positive or negative expression of VEGFR2.

**Supplementary Video**

**Supplementary Video 1. iEC migration in VEGF gradient.** C1-2 iEC migration in the channels with a VEGF gradient.

**Supplementary Video 2. iECs migration without VEGF gradient.** C1-2 iEC migration in the channels without a VEGF gradient.

**Supplementary Tables**

**Supplementary Table 1** hiPSC lines used in the study

| Cell Line | Source | Gender | Age | Generation Method | Cell origin and approvals |
| --- | --- | --- | --- | --- | --- |
| C1-2^1^ | Fibroblast | Male | Newborn | Episomal Vector | ATCC (CRL-2097) |
| 6.2^2^ | Cord Blood CD34+ Cells |  | Newborn | Episomal Vector | With the approval of  The Johns Hopkins University Institutional Review Board  the Johns Hopkins University and Institutional Stem Cell Research Oversight Committee |
| 1159^3^ | Skin Fibroblast | Female | 34 | mRNA | With the approval of the Columbia University Institutional Review Board and the Columbia University Embryonic Stem Cell Committee. |

**Supplementary Table 2** Antibodies used in the study

| Antibody | Source | Catalog # | Purpose | Host Species and Reactivity | Concentration |
| --- | --- | --- | --- | --- | --- |
| AlexaFluor 488 | ThermoFisher Scientific | A11008 | IF | Goat anti-rabbit | 1:1000 |
| HK1 | Cell Signaling Technology | 2024 | WB | Rabbit anti-human | 1:1000 |
| HK2 | Cell Signaling Technology | 2867 | WB | Rabbit anti-human | 1:1000 |
| PFKFB3 | Proteintech | 13763 | WB | Rabbit anti-human | 1:1000 |
| PFKP | Cell Signaling Technology | 8164 | WB | Rabbit anti-human | 1:1000 |
| PKM 1/2 | Cell Signaling Technology | 3190 | WB | Rabbit anti-human | 1:1000 |
| GAPDH | Cell Signaling Technology | 5174 | WB | Rabbit anti-human | 1:1000 |
| Beta Actin | Abcam | ab8226 | WB | Mouse anti-human | 1:1000 |
| CD309-PE | BD Biosciences | 560494 | FC | Mouse anti-human | 20 uL/ 10^6^ cells |
| Phospho-VEGFR2 (Tyr996) | Cell Signaling Technology | 2474 | WB | Rabbit anti-human | 1:1000 |
| Phospho-VEGFR2 (Tyr1175) | Cell Signaling Technology | 3770 | WB | Rabbit anti-human | 1:1000 |
| CD31-PE | BD Biosciences | 555446 | FC | Mouse anti-human | 20 uL/ 10^6^ cells |

**References**

1. Wen Z, Nguyen HN, Guo Z, Lalli MA, Wang X, Su Y, Kim NS, Yoon KJ, Shin J, Zhang C, Makri G, Nauen D, Yu H, Guzman E, Chiang CH, Yoritomo N, Kaibuchi K, Zou J, Christian KM, Cheng L, Ross CA, Margolis RL, Chen G, Kosik KS, Song H and Ming GL. Synaptic dysregulation in a human iPS cell model of mental disorders. *Nature*. 2014;515:414-8.

2. Burridge PW, Thompson S, Millrod MA, Weinberg S, Yuan X, Peters A, Mahairaki V, Koliatsos VE, Tung L and Zambidis ET. A universal system for highly efficient cardiac differentiation of human induced pluripotent stem cells that eliminates interline variability. *PLoS One*. 2011;6:e18293.

3. Sui L, Danzl N, Campbell SR, Viola R, Williams D, Xing Y, Wang Y, Phillips N, Poffenberger G, Johannesson B, Oberholzer J, Powers AC, Leibel RL, Chen X, Sykes M and Egli D. beta-Cell Replacement in Mice Using Human Type 1 Diabetes Nuclear Transfer Embryonic Stem Cells. *Diabetes*. 2018;67:26-35.
