# Supplementary material for: Intrinsic epigenetic control of angiogenesis in induced pluripotent stem cell-derived endothelium regulates vascular regeneration
Source: NPJ Regen Med. 2022 May 12;7:28. doi: 10.1038/s41536-022-00223-w (PMC9098630; doi:10.1038/s41536-022-00223-w)
Supplement: Supplementary file 4 — REPORTING SUMMARY [file 41536_2022_223_MOESM4_ESM.pdf]

nature portfolio

Corresponding author(s): Professor Sharon Gerecht

Last updated by author(s): Apr 7, 2022

Reporting Summary

Nature Portfolio wishes to improve the reproducibility of the work that we publish. This form provides structure for consistency and transparency in reporting. For further information on Nature Portfolio policies, see our Editorial Policies and the Editorial Policy Checklist.

Statistics

For all statistical analyses, confirm that the following items are present in the figure legend, table legend, main text, or Methods section.

n/a	Confirmed	
☐	☒	The exact sample size (n) for each experimental group/condition, given as a discrete number and unit of measurement
☐	☒	A statement on whether measurements were taken from distinct samples or whether the same sample was measured repeatedly
☐	☒	The statistical test(s) used AND whether they are one- or two-sided<br>*Only common tests should be described solely by name; describe more complex techniques in the Methods section.*
☒	☐	A description of all covariates tested
☒	☐	A description of any assumptions or corrections, such as tests of normality and adjustment for multiple comparisons
☐	☒	A full description of the statistical parameters including central tendency (e.g. means) or other basic estimates (e.g. regression coefficient) AND variation (e.g. standard deviation) or associated estimates of uncertainty (e.g. confidence intervals)
☐	☒	For null hypothesis testing, the test statistic (e.g. F, t, r) with confidence intervals, effect sizes, degrees of freedom and P value noted<br>*Give P values as exact values whenever suitable.*
☒	☐	For Bayesian analysis, information on the choice of priors and Markov chain Monte Carlo settings
☒	☐	For hierarchical and complex designs, identification of the appropriate level for tests and full reporting of outcomes
☒	☐	Estimates of effect sizes (e.g. Cohen's d, Pearson's r), indicating how they were calculated

Our web collection on statistics for biologists contains articles on many of the points above.

Software and code

Policy information about availability of computer code

Data collection	Confocal Images: Zeiss Laser Scanning Microscope 780 confocal microscope.<br>Timelapse microscopy: inverted Nikon Eclipse Ti Microscope.<br>Seahorse Assay: Seahorse XF24 Analyzer<br>Flow Cytometry: BD FACSCaliber<br>Western Blot Images: ChemiDoc XRS+<br>RT qPCR: QuantStudio5
Data analysis	Statistical Analysis: Prism GraphPad9<br>Spheroid Quantification/Filopodia Quantification/Timelapse Quantification/Fin Regeneration: ImageJ<br>Seahorse Assay: Wave Desktop<br>Flow Cytometry: FCS Express 6 Flow<br>Wester Blot: Image Lab

For manuscripts utilizing custom algorithms or software that are central to the research but not yet described in published literature, software must be made available to editors and reviewers. We strongly encourage code deposition in a community repository (e.g. GitHub). See the Nature Portfolio guidelines for submitting code & software for further information.

March 2021

Data

Policy information about availability of data

All manuscripts must include a data availability statement. This statement should provide the following information, where applicable:

- Accession codes, unique identifiers, or web links for publicly available datasets
- A description of any restrictions on data availability
- For clinical datasets or third party data, please ensure that the statement adheres to our policy

All data generated or analyzed during this study are included in this published article (and it's supplementary information files).

Field-specific reporting

Please select the one below that is the best fit for your research. If you are not sure, read the appropriate sections before making your selection.

☒ Life sciences ☐ Behavioural & social sciences ☐ Ecological, evolutionary & environmental sciences

For a reference copy of the document with all sections, see nature.com/documents/nr-reporting-summary-flat.pdf

Life sciences study design

All studies must disclose on these points even when the disclosure is negative.

Sample size	Spheroids, Seahorse Assay, Microfluidic Experiment, RT q-PCR, and flow cytometry all were conducted with N=3, with each N representing 3 separate differentiations.<br>ChIP PCR was performed for N=2 based on manufactures recommendation.<br>Zebrafish experiments were conducted for N=3 with each representing different injection experiments.
Data exclusions	No data was excluded from this manuscript
Replication	All experiments included both biological and technical replicates, with all data included in this manuscript.
Randomization	Zebrafish were randomly selected for experimental or control groups.
Blinding	No blinding was used in this study.

Reporting for specific materials, systems and methods

We require information from authors about some types of materials, experimental systems and methods used in many studies. Here, indicate whether each material, system or method listed is relevant to your study. If you are not sure if a list item applies to your research, read the appropriate section before selecting a response.

Materials & experimental systems

n/a	Involved in the study	
☐	☒	Antibodies
☐	☒	Eukaryotic cell lines
☒	☐	Palaeontology and archaeology
☐	☒	Animals and other organisms
☒	☐	Human research participants
☒	☐	Clinical data
☐	☐	Dual use research of concern

Methods

n/a	Involved in the study	
☒	☐	ChIP-seq
☐	☒	Flow cytometry
☒	☐	MRI-based neuroimaging

Antibodies

Antibodies used	See Supplementary Table 2
Validation	Validation was performed by the manufacturers of each antibody.

Eukaryotic cell lines

Policy information about cell lines

Cell line source(s)	HUVECs, Promocell. C1-2 iPSCs, 6.2 iPSCs, and 1159 iPSCs were all provided by collaborators, citation sin text.
Authentication	All iPSCs were karyotyped yearly.
Mycoplasma contamination	All cell liness are cytoplasma tested quarterly.
Commonly misidentified lines (See ICLAC register)	N/A

Animals and other organisms

Policy information about studies involving animals; ARRIVE guidelines recommended for reporting animal research

Laboratory animals	See methods "Zebrafish maintenance and husbandry" section.
Wild animals	N/A
Field-collected samples	N/A
Ethics oversight	Animal studies described herein were performed per the National Institutes of Health (NIH) Office of Laboratory Animal Welfare (OLAW) policies regarding studies conducted in vertebrate species.

Note that full information on the approval of the study protocol must also be provided in the manuscript.

Dual use research of concern

Policy information about dual use research of concern

Hazards

Could the accidental, deliberate or reckless misuse of agents or technologies generated in the work, or the application of information presented in the manuscript, pose a threat to:

No	Yes	
☒	☐	Public health
☒	☐	National security
☒	☐	Crops and/or livestock
☒	☐	Ecosystems
☒	☐	Any other significant area

Experiments of concern

Does the work involve any of these experiments of concern:

No	Yes	
☒	☐	Demonstrate how to render a vaccine ineffective
☒	☐	Confer resistance to therapeutically useful antibiotics or antiviral agents
☒	☐	Enhance the virulence of a pathogen or render a nonpathogen virulent
☒	☐	Increase transmissibility of a pathogen
☐	☐	Alter the host range of a pathogen
☒	☐	Enable evasion of diagnostic/detection modalities
☒	☐	Enable the weaponization of a biological agent or toxin
☒	☐	Any other potentially harmful combination of experiments and agents

Flow Cytometry

Plots

Confirm that:

☐ The axis labels state the marker and fluorochrome used (e.g. CD4-FITC).

☐ The axis scales are clearly visible. Include numbers along axes only for bottom left plot of group (a 'group' is an analysis of identical markers).

☒ All plots are contour plots with outliers or pseudocolor plots.

☒ A numerical value for number of cells or percentage (with statistics) is provided.

Methodology

Sample preparation	See Methods "Flow Cytometry" section
Instrument	BD FACSCaliber
Software	BD FACSDiva was used to collect data, FCS Express 6 Flow was used to analyze data
Cell population abundance	N/A
Gating strategy	Events were first gated for presence of single events, followed by exclusion of dead cells, then positive and negative expression of VEGFR2.

☒ Tick this box to confirm that a figure exemplifying the gating strategy is provided in the Supplementary Information.
